# Supplementary material for: Electrophysiological responses of relatedness to consecutive word stimuli in relation to an actively recollected target word
Source: Sci Rep. 2019 Oct 10;9:14514. doi: 10.1038/s41598-019-51011-4 (PMC6786994; doi:10.1038/s41598-019-51011-4)
Supplement: Supplementary file 1 — Supplementary Information [file 41598_2019_51011_MOESM1_ESM.pdf]

Supplementary Information

Electrophysiological responses of relatedness to consecutive word stimuli in relation to an actively recollected target word

Karen Dijkstra, Jason Farquhar, Peter Desain

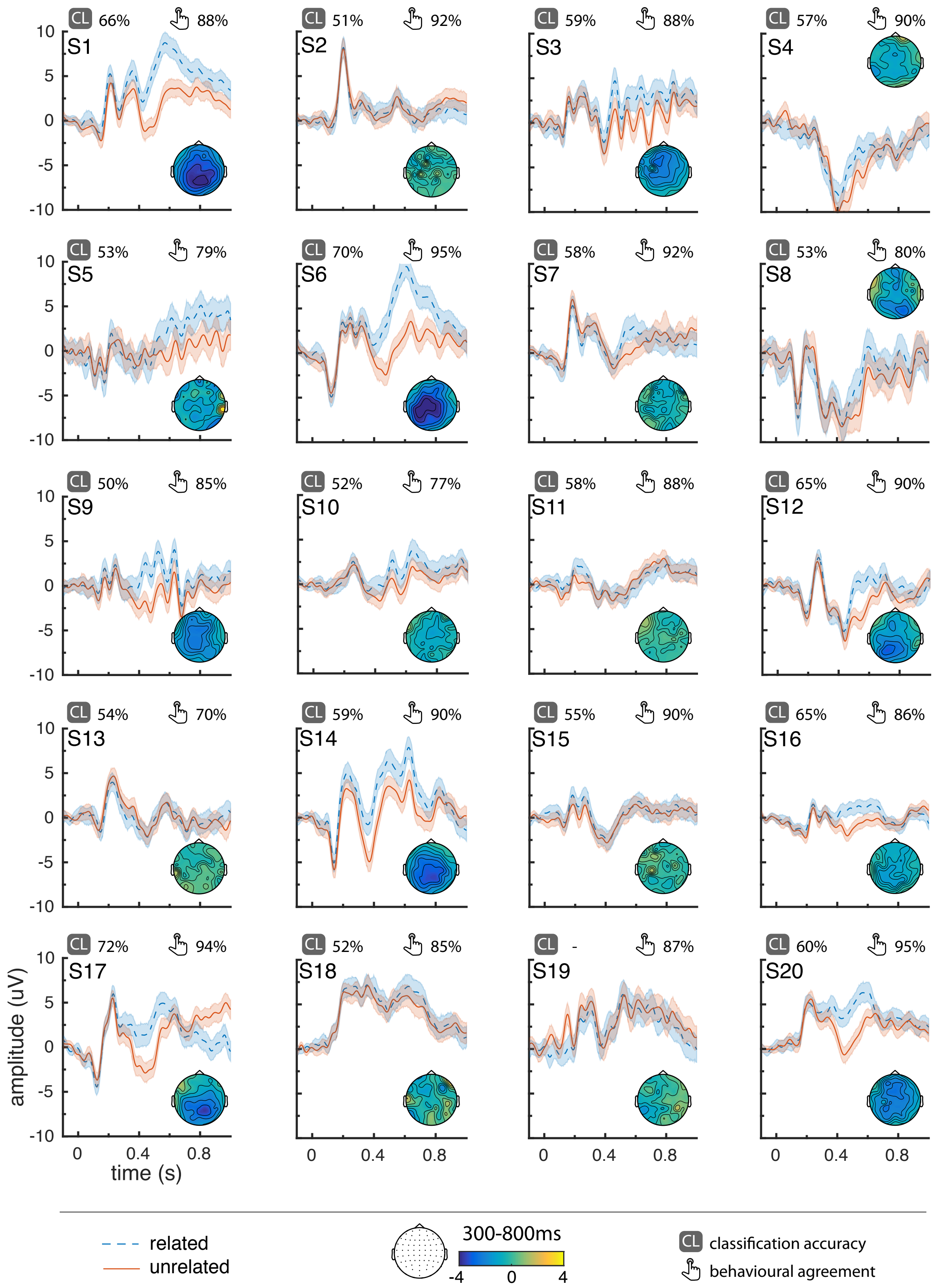

Supplemental Figure S 1. Individual ERPs with shaded 95% bootstrapped confidence interval of the mean. Spatial topographies from 300-800ms are included. Classification rates and behavioural agreement with the ground truth labels are specified for each subject.

Supplementary Methods: Description of stimuli

Stimulus words were obtained from the Leuven association dataset (Deyne and Storms; 2008), and the CELEX dutch wordform database (Baayen et al.; 1995; celex.mpi.nl). The Leuven dataset is a Dutch word-association database that consists of cue-words and responses from people who were asked to specify (up to) three words that a given cue-word brought to mind. The database is a raw collection of the human responses, and can contain misspelled words or nonsensical responses. To construct a sensible stimulus, set we filtered the responses in this database to contain only headwords (i.e., how a word occurs in the dictionary), by matching it against a CELEX list of words with the 'headword' property.

The Leuven cue-words could then be used as targets, and the responses to these cue-words as related probes. To determine how targets and probes could best be allocated to trials, we constructed a trial framework such that 50% of trials contained ten probes, and the remaining trials contained an equal distribution of one to ten probes. We ensured that the first and final (9th and 10th) positions contained an equal number of related and unrelated probes. The remaining probe positions (2nd through 8th) were designated to contain a related probe with 1/3 probability, while ensuring no two related probes occured consecutively.

Having now specified the number of related and unrelated probes required for a given trial, we could now assign targets (i.e. Leuven cue-words) with a sufficient number of related probes (i.e., Leuven responses) to each trial. To avoid repetition effects, probes that were used in one trial, did not occur in any other trial as a probe. However, some probes did also occur as a target. In such cases, the trial in which it occurred as a probe would be presented earlier in the experiment than its occurrence as a target, where possible. Unrelated probes were drawn from the previously mentioned CELEX head-word list, but now with the already selected related probes and targets removed. For each trial, we made sure that the selected unrelated probes had not been given as a response to the cue that corresponded to that trial's target, according to the Leuven database.

Furthermore, to make sure the unrelated probes had similar word length and word frequency, we first sampled a subset of CELEX words to match the distribution of word lengths to that of the already selected related probes. Then, when assigning unrelated probes, these probes were sampled to match the distribution of word frequencies of the related probes. Across all probes, the mean (log) word frequency was 1.50 (sd = 0.65) for related, and 1.49 (sd = 0.63) for unrelated probes. This resulted in average word lengths of 5.68 (sd = 1.98) for related, and 6.13 (sd = 1.92) for unrelated probes.

Finally, the resulting trials were manually checked to remove any issues (e.g., probes assigned as unrelated being related by chance).

The trials were then shuffled per participant, in such a way that probes that were also targets, first appeared as a probe before they did as a target. Note: due to some three-way connections across trials this was not *always* possible.

Supplementary Methods: List of stimuli

Below, a complete list of stimuli presented in the experiment. Targets are **bolded**, and related probes are underlined.

slapen - schoot - dromen - edel - nacht
wandelen - koel - chip - park - zuiver
verpleegster - wand
hoofd - denken - omgeven - hersenen - grootte - engel
zilver - juweel - vijand - vertrek - ketting - heuvel - body - genegenheid - druppel
leger - soldaat - strak - belonen - tank - ark - geweer - weten
molen - graan - aurora - wind - bekken - meel
banaan - aap - emmer - zes - krom
sport - tennis - apart - openen - voetbal - aangifte - zwemmen - aanmoedigen - zweet - buik - lopen
snijden - bodem - scherp - mate - mes - volk - bloed - aankomen - cijfer - atelier - vlees
vinger - bouwer - weigeren - erf - duim - behang - gedrukt - winst - dank - huidig - wijzen
berg - top - steun - bui - natuur - flits - buit
wandelstok - basis - zijde - jongere - wandelen - badkuip - opa - aula - heten - bergen - bonken
gordel - veiligheid
revolver - charme - bot - roman - cowboy - grijns - staf - droevig - zeven - bestuur - moord
handtas - wilde - rommel - stem - leer - beest - geld - dertig - veranderen - vrouw - belofte
hamer - kloppen - boeg - getuigenis - gewijd - pogen - ruim - komen - gade - spijker - verenigen
keuken - tafel
toetsen - leren - afkeuring - bezocht - verhouding - stress - begeerte - veel - computer - bel - examen
bar - drinken - meemaken - tap - bepalen - kruk - zingen - behoefte - gedaan
zitten - staan - poging - consul - bank - dunne - vlucht - totaal
sigaret - roken - belijden - ongezond - armoedig - kanker - eindeloos - elleboog - beschouwen - tabak - sluiten
gewicht - kilo - durven - symbool - brein - weegschaal - bannen - jagen - juli - barkeeper - zwaar

aarde - simpel - beverig - bakje - bol - bevoegde - slag - planeet - lijn - wereld - keren
dierenarts - erkenning - stuur - knaap
blazen - beklag - zakken - handeling - ballon - gids - conceptie
koningin - kust - paleis
boek - bibliotheek - afweer - ontspanning - achterste - spannend - baron - roman - gebied - waar
hoorn - driftig - telefoon - lijf - blazen - evenwicht
boter - smeren - autobus - vet - autonomie - bakken - avenue
touw - eeuwigheid - koord - anus - trekken - jaar - arend - knoop - leeg - klimmen - boord
naaimachine - naald - conditie - stikken - betoging - wet - emotie - automaat - banning - garen - boerin
vuil - modder - blos - afval
vrouw - genoemd - mooi - afwegen - man - afbeelding - moeder - geleid - afnemen - erg - kind
pruim - integratie - lichaam - gang - vrucht - actief - paars - bazin - barst - pit - beslissend
stofzuiger - gezet - vuil - arena - armoe - stof - rijke - lawaai - binden - golf - tapijt
beeld - noemen - schaal - plaat - losmaken - kunst - beheerder - foto - meest - standbeeld - juist
bijl - brandstof - dulden - opvallen - proeven - buisje - bindmiddel - binnenste - lijk - dozijn - hakken
bliksem - los - assistentie - regen - bijbels - donder - relatief - hut - brullen - onweer - adem
oud - afbreken - afmeting - grootouders - afgrond - beleid - jong - adoptie - oma - anoniem - versleten
fruit - peer
pop - gezegd - speelgoed - aangeven - meisje - bezinning - spelen
kano - uitleggen - verbinding - doorgang - gekleed - dreun
oester - schelp - attent - parel - activa - akker - slijm - vorig - paniek - bezoek - zout
kleur - zonde - ruzie - hees
fabriek - werken - genieten - werk - abstractie - rook - jongeman - arbeider
boom - tak - gering - gereed - letten - stam - westen
cowboy - bijlage - zaal - hoed - kil - oktober - neiging
tand - balsem - speciaal - broer - leveren - concern - tandarts - opvatting - gebit - verlenen
proper - net - bestand - bevalling - schoon - leven - bizar - bloeddruk - diplomaat - netjes - dienen
rechter - toga - eigen - gevel - ban - advocaat - reeks - rechtbank - gerust - belast
peer - appel - voorzien - brons
staart - bidden - paard - haat - hond - atmosfeer - kat - amulet - leerling - luid - lang
panda - karakter - kern - beer - informatie - kwartier - bamboe - gaande - boa - deftig
handdoek - doorbreken - normaal - drogen - zaak - afwas - lieden - revolutie - beding - droog - diepte
pasta - hoop - knie - kosten - schok - wagen - spaghetti - beleven - burger - stoten - saus
wol - brandend - schaap - ballet - onverwacht - relatie - trui - maal - pakken - breien - bits
reis - braken - drager - vak - hertog - verstand - voldoen - fabriek - klacht - ver - firma
kleerkast - kleren - afwijking - aandoen - slaapkamer - bemanning - achterhoede - kledij - meneer - moment - kapstok
dief - bezield - vlag - oprit - cacao - overgaan - vermogen - storten - bijval - camping - stelen
hond - gevraagd - werking - huisdier - berk - smal - ontlenen - handje - abrupt - blaffen - bestelwagen
inkt - vlek - bokser - trek - luiden - mand - vulpen - werker
munt - thee - buffer - mus
lucht - vliegtuig - adres - ademen - lichten - kaal - min - berouw - laten - wolk - twintig
boerderij - boer - geestelijke - koe - baken - varken
teen - poos - stimuleren - oever - radio - vrede - gunst - wijd - boze - voet - bushalte
parachute - rollen - ego - spannen - inclusief - donkergroen - ruw - spiegel - continu - springen - streven
clown - grappig - bitter - gauw - circus - compleet - leunen - lachen - ingaan
exotisch - cocktail - ader - zon - bedragen - ananas - dichtbij - eiland - aanpak - barsten
tas - grap - buikpijn - zak - visie - wonder - puur - bewonderenswaardig
computer - scherm - schouder - toetsenbord
noordpool - zuidpool - fort - rapport
zien - kijken - kraken
punt - komma - haven - potlood - belang - zin - geest - axioma - dekken - bewijzen - asiel
scheermes - schuim - klasse - bukken - baard - balie - snijden - artiest - doos - benoemen - gebogen
akker - beschut - veld - aroma - eis - kruipen - land - gemiddeld - doen - dapper - landbouw
hand - vinger - wil - norm - genoegen - beraad
garnaal - zee - aanzienlijk - functie - lekker - aanleren - tomaat - baseren - vis - ruimte - grijs

slim - intelligent - weggaan - behendig - dom - gemak
zwaan - uiting - blok - kom - zenden
bezem - heks - bil - bedaren - omvang - partij - benzine - steel - datje - vaak - vegen
wesp - steek - liegen - bedrog - bij - aanbod - steken - asbak - orde - anjer - angel
tram - anima - sporen - duif - atleet - stad - kist - behoren - spoor - krant
televisie - nieuws - dubbel - film - tamelijk - programma - exact
wiel - kar - benul - voornaam - band - bondig
rat - vies - genomen - riool - fris - staart - doden - eeuwig - hoeven - muis - bewegen
meloen - oranje - type - dorp - zomer - balans - sappig - bouw - leider - rond - boeien
drinken - dorst - citeren - cola - achtergrond - beroep - lenen - acceptatie - alcohol - verder - glas
raam - bon - inzien - twijfel - venster - bronzen - commando - vullen - boosheid - proef - buiten
wild - hert - moed
hek - afsluiting - verte - baret - omheining - niveau - liggend
bril - westers - volwassen - zuster - lezen - fijn - bewoner - montuur - long - boel - glazen
kooi - gevangen - dragen - museum - week - leeuw - loop - gevangenis
muur - hoog - bestaan - ambitie - cement - alarm - affaire - afzet - baksteen - balk - hard
jeuk - krabben - mens - uitslag - ideologie - bast - elf - beleg - mug
post - postbode - arsenaal - brievenbus - dode - zwarte - bejaard - aspirine - postzegel - degelijk
tuin - behoud - planten - gezag - gave
priester - bouillon - tevreden - bruto - bestuursrecht - kruis - voedsel
lade - kast
kampvuur - warmte - druif - gezelligheid - drie - gezellig
handschoen - koud - contact - warm - heer - winter - agent - sneeuw - krijgen - hand - afkeer
veel - bankje - weinig - huren - massa - barman - meer
taart - ergeren - boef - slagroom - ford - aanpassen - keel - verjaardag
station - trein - democratie - beoordeling - wachten - rol - perron - formule
oor - vinden - later - horen - geval - luisteren - baai - affectie - muziek - een - geluid
knol - wortel - pagina - jeugd - gezocht - grond - dek - sprake - fors - aardappel - lot
baas - vroeger - streng - leggen - chef - boog - beurs - bureau - orgaan
verkeer - facet - file - kopje - ziekte - duidelijk - bezwaar - drukte - ding - vereniging - druk
geweer - schieten - afgemeten - middel - kogel - daad - as - jacht - aanplant - bond - dood
koe - weide - leraar - akte - gras - context - kalf - beheer - bestuurlijk - wei - sprong
duivel - slecht - bijten - bloedig - hangen - blank - dun - hel - vast - satan - vorm
schild - positie - bereik - deugd - ridder - baarmoeder - cent - pad - diep - bescherming - stand
spijker - kop - baas
kanarie - deeg - vuist - kooi - verstaan - zuiden - fluiten - boeten - border - bod
glijden - slee - juni
koning - koningin - eindje - dominee - kroon - rustig - troon - alfa
broek - rok - ouder - noodzaak - strijd - tien - grote - rits - sterk - jeans - redden
blok - billijk - collectie - bindend - beton - vormen - bisdom - studeren - bacon - breedte - been
uniform - leger - aanstelling - afkomst - politie - half - school - aanvraag - zit - blauw - auteur
peper - kwaad - gedaante - pikant - denkend - titel
bok - jasje
vulkaan - lava - azijn - gaan - bede - huid - uitbarsting - doel - gast - berg - negeren
schoorsteen - gezin - toon - zwart - formeel - isoleren - sinterklaas - oude - kijker - roet - begrip
tang - duiden - botsing - ijzer - goed - aquarium - hoek - gereedschap - beestje - werktuig - best
robot - metaal - zak - atoombom - eigenschap - toekomst - gebruik - machine - begerig
engel - hemel - cursus - arrestatie - lief - ambt - duivel - madame - duren - god - bladzijde
das - feest - bak - hemd - taak - dal - leiden - kostuum - kader - pak - oog
trouwen - ring - alfabet - antenne - kerk - eng - dagboek - liefde - begaan - jurk - arrogant
kapitein - gebrek - vijfde - schip - tocht - stevig - noorden - vaag - bekleed - matroos - rij
badkamer - douche - grens - wassen - vier - bad - model - aanzien - afslag - handdoek - ervaren
helm - graad - brommer - expert - motor - bijstand - collega - oor - blom - veilig - externe
ontbijt - meten - boterham - assistent - koffie - bewind - brood - bedrijvigheid - kennis
pad - groei - kikker - sturen - glibberig - astma - andijvie - blik - vijver - angina

vonnis - gerecht - verband - rechter - beitel - beklemming - blij - merken - nerveus - straf - bakkerij
bom - explosie - afhouden - beiden - wensen - oorlog - bende - ontploffing - breed - binnenlands - geweld
yoghurt - wit - officier - melk - afhalen - fruit - voelen - suiker - avontuur - aankoop - gezond
eiland - vrezen - dimensie - onbewoond - kleden
eiwit - kip - bootsman - eigeel
beha - kant - helder - ondergoed - berber - klagen - barok
knopen - opzicht - touw - zetten - jas - debat
knippen - schaar - praten - enorm - papier - enig - hanteren - kapper - dalen - haar - afname
pot - deksel - kiezen - ketel - fase - betoog - agenda - aarde - berekenen - afdronk - plant
gitaar - buffet - defensie - interessant - schuin - snaar - opmerking - prins
chips - broche - slepen - paprika - cilinder
opstaan - intiem - inwoner - elite - vroeg - den - bosrand - eens - kring - wekker - domein
rad - fel - wiel - barbaars - heet - kermis - koffer - zoon - draaien - klas - fortuin
tafel - tafelkleed - verwijderen - rede - nodig - poot - binnenland - nek
omelet - ei - alibi - grof - geel - allergisch - pan - dag - ogenblik - spek - abdij
dieet - beige - stijgen - experiment - beloven - citroen - honger - duwen - bedolven - mager - nota
papegaai - boeddha
aas - gier - benadering - worm - aanwending - vissen - droom - absurd - kaarten - aapje - hengel
schakelaar - geloven - lamp - baden - brengen - chaos - knop - beeld - aan - atoom - elektriciteit
ruiken - geur - grondslag - parfum - aangeschoten - huwelijk - stank - missen - eten - afwezig - bloem
divan - begeven - liggen - anker - zetel - verlies - rusten - brutaal - ambassade - reden - slapen
hak - beetje - winkel - erkend - bijl - verzet - beha - heffen - kwestie - hiel - vergelijken
weg - baan - schaduw - genie - broekspijp - bouquet - dialect - worden - mama - bazaar - straat
magneet - nood - wenden - aantrekkingskracht - hoesten
anker - oosters - doolhof
vuist - circuit - kapot - prof - maan - bestuurslid - naakt - vechten - cyclus - mei - verslag
trap - gebed
kraam - nadruk - gretig - waarneming - koers - provincie - riks - pastoor - blaas - markt - buis
kogel - rijzen - pistool
naaien - klaar - naaimachine - stap - kleding
ladder - inhoud - geniaal - dak - champagne - boezem - bas - pop - gigantisch - trede - onzeker
microscoop - onderzoek - achttiende - acceptabel - arriveren - dame - klein - gat - wetenschap - stukje - biologie
venkel - crisis - kruid - zuchten - betaald - soep - gevoel - groen - aartsbisschop - groente - gevaar
dessert - taart - fantastisch - bedachtzaam - chocolade - factor - beide - zoet - bloei - pudding - naam
instrument - hoge - gitaar - dicht - viool - glanzen - trompet - globaal - kamer - bereikbaar - piano
neus - animo - gezicht - beter - ruiken - behouden - glimlach - zakdoek - beoordelen - beroemd - groot
ridder - beugel - doctor - harnas - casino - doorn - zwaard - moeten - blaadje - flink - prinses
ketting - blinde - zaag - breuk - hals - keer - pers - benoeming - ernst - rode - goud
schaken - bochel - moeilijk - cabine - plicht - joods - koning - oorzaak - vol - spel - stroom
tijd - stijl - horloge - afweten - boekje - waarheid - regel - klok - inspanning - bederf - uurwerk
bikini - zwembad - bevriend
tijger - geografisch - wild - typisch - geheel - uitgaan - gevaarlijk - badwater - begroeid - blouse - jungle
kussen - capitulatie
ondergronds - metro - afgunstig - beker - kelder - beroven - derde - reis - donker - bruikbaar - tunnel
sleutel - consumptie - wens - slot
lepel - apathie - vork - maand - bord - papa - bestek
speer - falen - actie - werpen - bewering - punt - raad - hebben - opgeven - wapen - verhaal
palet - hout - bestaand - analoog - schilderen - allereerst - verf - hulp - aardgas - schilder - album
veer - baren - zacht - dof - neger - pluim - banaal - vogel - argeloos - nadenken - schrijven
woestijn - kameel - beuk
wolf - janken
glibberig - alomvattend - vallen - enkel - ijs - angst - deel - glad - aardig - anarchie - slang
kluis - graaf - code - vloer - wang
bakken - held - tonen - bezetter - eisen - cake - prijs - oven - beul - bakker - heftig
alcohol - kennen - bier - behulp - wijn - afwijzing - drank - matig - dronken

bromfiets - front - jack - damp - vochtig
snavel - vangen - datum - eend - hek - helling - dij - dichter - geloof - terras - bek
chauffeur - mogen - auto - beginnen - bus - katholiek - afgewogen - taxi - aanhouden - afgelegen - rijden
snel - hoger - vlug - financieel - dor - diner - traag - hevig - bron - rap - bunker
geit - aanloop - bok - aanrecht - kaas - bevel - kort - dier - borst - boerderij
parlement - minister - atlas - bezoeker - regering - gedragen - ellende - politiek - afzender
lijm - hecht - kleven - haten
schorpioen - giftig - minuut - groeten - doodstil - verschil - raam - gif - colonne - concert - woestijn
zondag - haak - rust - attractie - bankier - anders - vrij - stil - bang - weekend - prachtig
rits - open - grazen
lucifer - vuur - feit - zwavel - baat - brand - aandeel - inzicht - borrel - afdalen - sigaret
politieagent - uniform - geheim - boete - bakermat - halen - bijzijn - docent
doorzichtig - slaaf
mooi - beurt - links - soort - mantel - borg - lelijk - uitspraak - rand - dreiging - knap
tandpasta - kost - gewoon - college - tandenborstel - flauw - poetsen - jong - bedwang - munt - zorgen
praten - boon - farao - gesprek - nauw - extra - echt - mond - aura - badjas - babbelen
geest - spook - mening - ziel - bordeaux - vals
baby - schattig - even - landen - apotheek - armslag - huilen - lepel - commandant - terecht - luier
tong - smaak - flat
dak - schoorsteen - opkomen
goud - zilver - alinea - hou - tikken - gelukkig - rijk - dekker - reactie - ervaring - duur
kabouter - paddestoel - begin - heersen - muts - belevenis - lied - amateur - tuin - grijpen - dwerg
team - cognac - sport - buigen - plek - groep - afspraakje - samen - abces - taal - ploeg
telefoon - bellen - lezer - nummer - bons
